# Mixed methods evaluation of handshake antimicrobial stewardship on adult inpatient medicine floors

**DOI:** 10.1017/ash.2023.465

**Published:** 2023-11-16

**Authors:** Elizabeth A. Neuner, Andrew Atkinson, Dan Ilges, Tamara Krekel, David J. Ritchie, Alice F. Bewley, Michael J. Durkin, Kevin Hsueh, Sena Sayood

**Affiliations:** 1 Department of Pharmacy, Barnes-Jewish Hospital, St. Louis, MO, USA; 2 Department of Internal Medicine, Division of Infectious Diseases, Washington University, School of Medicine in St. Louis, St. Louis, MO, USA; 3 Department of Pharmacy, Mayo Clinic Arizona, Phoenix, AZ, USA

## Abstract

**Objective:**

To evaluate the effects of handshake antimicrobial stewardship on medicine floors at a large tertiary care hospital.

**Design:**

Retrospective observational study.

**Setting:**

1,278-bed academic hospital.

**Patients:**

Adults admitted to non-ICU medicine services.

**Interventions:**

A handshake stewardship team consisting of an infectious diseases (ID) physician and pharmacist reviewed charts of patients receiving antimicrobials on medicine floors without a formal ID consult. Recommendations were communicated in-person to providers and acceptance rates were examined with descriptive statistics. Additional data regarding program perception among providers were obtained via surveys. Antibiotic usage trends were extracted from National Healthcare Safety Network Antimicrobial Use option data and evaluated using an interrupted time-series analysis pre- and post-intervention.

**Results:**

The overall acceptance rate of interventions was 80%, the majority being recommendations either to discontinue (37%) or de-escalate therapy (28%). Medical residents and hospitalists rated the intervention favorably with 90% reporting recommendations were helpful all or most of the time. There was a statistically significant decrease in vancomycin (78 vs 70 DOT/1,000 d present (DP), *p* = 0.002) and meropenem (24 vs 17 DOT/1,000 DP, *p* = 0.007) usage and a statistically significant increase in amoxicillin-clavulanate usage (11 vs 15 DOT/1,000 DP, *p* < 0.001). Overall antibiotic usage remained unchanged by the intervention, though pre-intervention there was a nonsignificant overall increasing trend while post-intervention there was a nonsignificant decreasing trend in overall usage. There was no change in in-hospital mortality.

**Conclusion:**

The addition of handshake stewardship with adult medicine services was favorably viewed by participants and led to shifts in antibiotic usage.

## Introduction

Handshake antimicrobial stewardship is a form of prospective audit and feedback characterized by the collaborative review of all antimicrobials by an infectious diseases (ID) physician and ID pharmacist followed by in-person rounds to provide feedback to prescribers.^1^ This intervention has been recognized as a “leading practice” by The Joint Commission and is highlighted in the Core Elements of Hospital Antimicrobial Stewardship defined by the Centers for Disease Control (CDC).^2,3^ Prior descriptions of this intervention in pediatric hospital settings have shown that handshake stewardship can have a sustained impact on reducing antimicrobial use without negatively impacting patient outcomes.^1^ In adult patient populations, literature often focuses on rounds in the intensive care units.^4–6^ Outside of intensive care units, there are some data supporting the use of “high intensity” or “academic detailing” strategies, similar to handshake stewardship, to reduce broad-spectrum antimicrobial use, but there are limited data on the applicability of this strategy more broadly in adult hospitals.^7–9^

Considering workload volume and engagement of key stakeholders, our antimicrobial stewardship program implemented handshake stewardship rounds in patients admitted to medicine floors. The purpose of this study was to describe the implementation of our service and evaluate its impact on antimicrobial use.

## Methods

Barnes-Jewish Hospital is a large, academic medical center (1,278 staffed beds) located in Saint Louis, Missouri that has had an antimicrobial stewardship program (ASP) since 1985. Core program components include prior authorization and 72-hour prospective audit and feedback of select antimicrobials, institution-specific guidelines for common infectious diseases, clinical decision support within the electronic health record (Epic®, Epic Systems Corporation, Verona, WI), and tracking/reporting of antimicrobial use data (additional details in Supplementary Table 1). From August 2019 to June 2021, dedicated program FTEs included 1.0 FTE of infectious diseases (ID) physician support and 2.0 FTE of ID pharmacist support. An additional 1.0 FTE ID physician support was added in July 2021 to support handshake antimicrobial stewardship.

Handshake stewardship was implemented on the thirteen medicine floors three days per week. The ∼250 medicine beds are covered by either one of twelve medicine teaching services (>150 internal medicine residents) or twelve medicine hospitalist services (>100 hospitalist physicians). Eleven units had both medicine teaching services and hospitalists providing care for patients on those floors, including three telemetry units for cardiology patients. The final two units housed patients cared for exclusively by the hospitalist service. Daily activities of the handshake stewardship service included reviewing all antimicrobials for patients admitted to a medicine service without a formal ID consultation split by the ID physician and ID pharmacist (∼1 h each). The ID physician and ID pharmacist then met to review cases and potential interventions (∼1 h). Reviews focused on general appropriateness of antimicrobial orders within the context of each individual patient. The team rounded in-person to discuss recommendations with the primary team providers (∼1.5 h). Following rounds, the ID physician and ID pharmacist documented recommendations in the pharmacy intervention system (∼0.5 h). Two days per week the handshake stewardship team reviewed medicine teaching service patients and one day per week hospitalist patients. Handshake stewardship rounds started in August 2021.

To account for unpredictable fluctuations in antibiotic use due to the COVID-19 pandemic, two years of pre- (August 2019–July 2021) and one year post- (August 2021–July 2022) implementation of the handshake stewardship service on medicine floors were evaluated. Antimicrobial use was measured in days of therapy (DOT) per 1,000 days present (DP) as reported to the CDC’s National Healthcare Safety Network Antimicrobial Use module. Data were aggregated for each month of the study periods. The antibiotic spectrum index (ASI) was calculated using an expanded and modified version developed by Gerber et al.^10,11^ Assessment of intravenous versus oral antibiotic usage was performed using the digestive DOT (dDOT) over total DOT (tDOT) metric as described by Moehring et al.^12^ Descriptive data on types of recommendations and acceptance rate were evaluated. Patient outcomes included length of stay and in-hospital mortality. Rates of *Clostridioides difficile* infection were also evaluated; testing was performed by a toxin EIA assay (Alere TOX A/B II, Abbott, Lake Bluff, IL) from August 2019 to February 15, 2022 and from February 16, 2022 to July 2022 testing was performed using a GDH & Toxin A/B combination assay (C. Diff Quik Check Complete® TechLab, Blacksburg, VA) with discordant results automatically reflexed to nucleic acid amplification (Cepheid Xpert C. difficile PCR Cepheid, Sunnyvale, CA). Feedback from medical residents and hospitalists was evaluated through surveys sent via email with two reminders over three weeks in December 2021 and November 2022, respectively (detailed survey questions in Supplementary Table 2).

This study was approved by the local institutional review board.

Intervention and survey data were examined using descriptive statistics. For comparisons of pre- and post-intervention groups, categorical data were compared by the *χ* ^2^ test and continuous variables with a Mann–Whitney test. Antibiotic usage per month and location were plotted along with a cubic spline smoother (with six equally spaced knots) to identify general temporal trends. Furthermore, an interrupted time-series analysis comparing both the immediate effect of the intervention (the intercept at time 0) and change of slope in DOT/1,000 DP pre-intervention to post-intervention was performed. Data were analyzed using R version 4.2.2 (R Foundation for Statistical Computing, Vienna, Austria).

## Results

The handshake stewardship service reviewed 7,410 patient charts, rounded 138 days, and made 1,558 recommendations (daily average of 54 charts and 11.3 recommendations, Table 1). The most common recommendations were to discontinue (37%), de-escalate (28%), or shorten/define duration (14%) of antimicrobials. The overall intervention acceptance rate was 80% but varied by intervention type from a high of 95% for dose optimization to a low of 58% for diagnostic-related recommendations.

**Table 1.** Description of handshake antimicrobial stewardship interventions

Number of charts reviewed	7410
Number of interventions	1558
Mean no. interventions/day	11.3 ± 4.9
Type of Antimicrobial Stewardship Recommendation	
Discontinue	573 (37%)
De-escalate^a^	437 (28%)
Shorten/define duration	216 (14%)
Diagnostic related	83 (5%)
Broaden coverage/address absent therapy	61 (4%)
Route change intravenous to oral	55 (4%)
Recommend infectious diseases consult	46 (3%)
Dose optimization	39 (2%)
Monitoring related	26 (2%)
Other^b^	22 (1%)
Antimicrobial/Antimicrobial class intervened upon^c^	
Ceftriaxone	352 (23%)
MRSA agents^d^	277 (18%)
Cefepime	244 (16%)
Carbapenem	106 (7%)
Metronidazole	99 (6%)
Fluoroquinolones	98 (6%)
Azithromycin	66 (4%)
Amoxicillin and Amoxicillin-clavulanate	44 (3%)
Tetracyclines	43 (3%)
Ampicillin-sulbactam	37 (2%)
Piperacillin-tazobactam	30 (2%)
Oral cephalosporins	26 (2%)
Antiviral agents	26 (2%)
Antifungal agents	25 (2%)
Other^e^	102 (7%)
Recommendation accepted, overall	1254 (80%)
Discontinue	477/573 (83%)
De-escalate	333/437 (76%)
Shorten/define duration	184/216 (85%)
Diagnostic related	48/83 (58%)
Broaden coverage/address absent therapy	51/61 (84%)
Route change intravenous to oral	42/55 (76%)
Recommend infectious diseases consult	37/46 (80%)
Dose optimization	37/39 (95%)
Supportive care/monitoring	23/26 (88%)
Other^b^	22/22 (100%)

Note. MRSA, methicillin-resistant *Staphylococcus aureus.*

a 223/437 (51%) De-escalation recommendations included a recommendation to switch from an IV agent to a PO agent with a narrower spectrum of activity.

b Other antimicrobial stewardship recommendation types included 13 lengthen duration of therapy, 8 drug interaction or contraindication -related, 1 allergy clarification.

c Multiple agents were intervened upon in 18% of overall recommendations; percentages do not add up to 100%.

d MRSA agents included ceftaroline, daptomycin, linezolid, and intravenous vancomycin.

e Other agents included 22 clindamycin, 21 sulfamethoxazole-trimethoprim, 16 ampicillin, 14 nitrofurantoin, 11 cefazolin, 5 aztreonam, 4 oral vancomycin, 2 atovaquone, 1 baricitinib, 1 dexamethasone, 1 fidaxomicin, 1 fosfomycin, 1 hydroxychloroquine, 1 oxacillin, and 1 penicillin.

**Figure 1.**
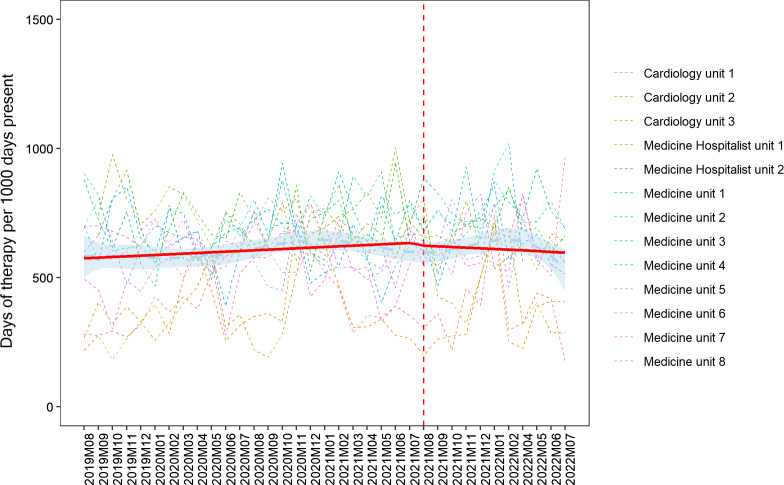
Antibiotic usage by month and location (dashed); intervention start dashed red line; overall cubic spline smoother shown (six knots, light blue/dashed) with 95% confidence intervals shaded, and average consumption from the predicted distribution of the interrupted time series segmented regression model (red/solid, interrupt at August 2021).

**Figure 2.**
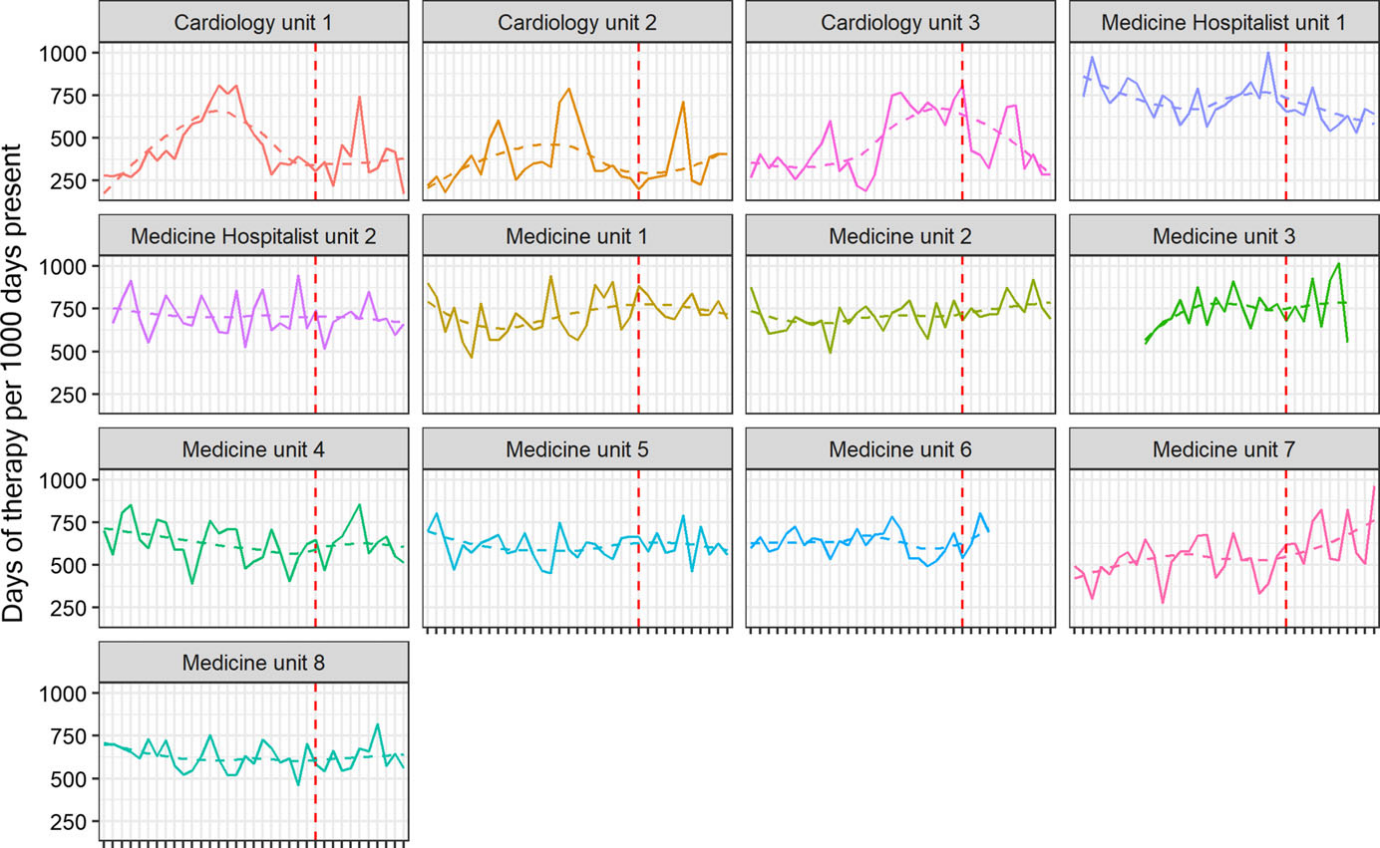
Monthly antimicrobial use by individual medicine unit; nonparametric smoother shown dashed.

**Figure 3.**
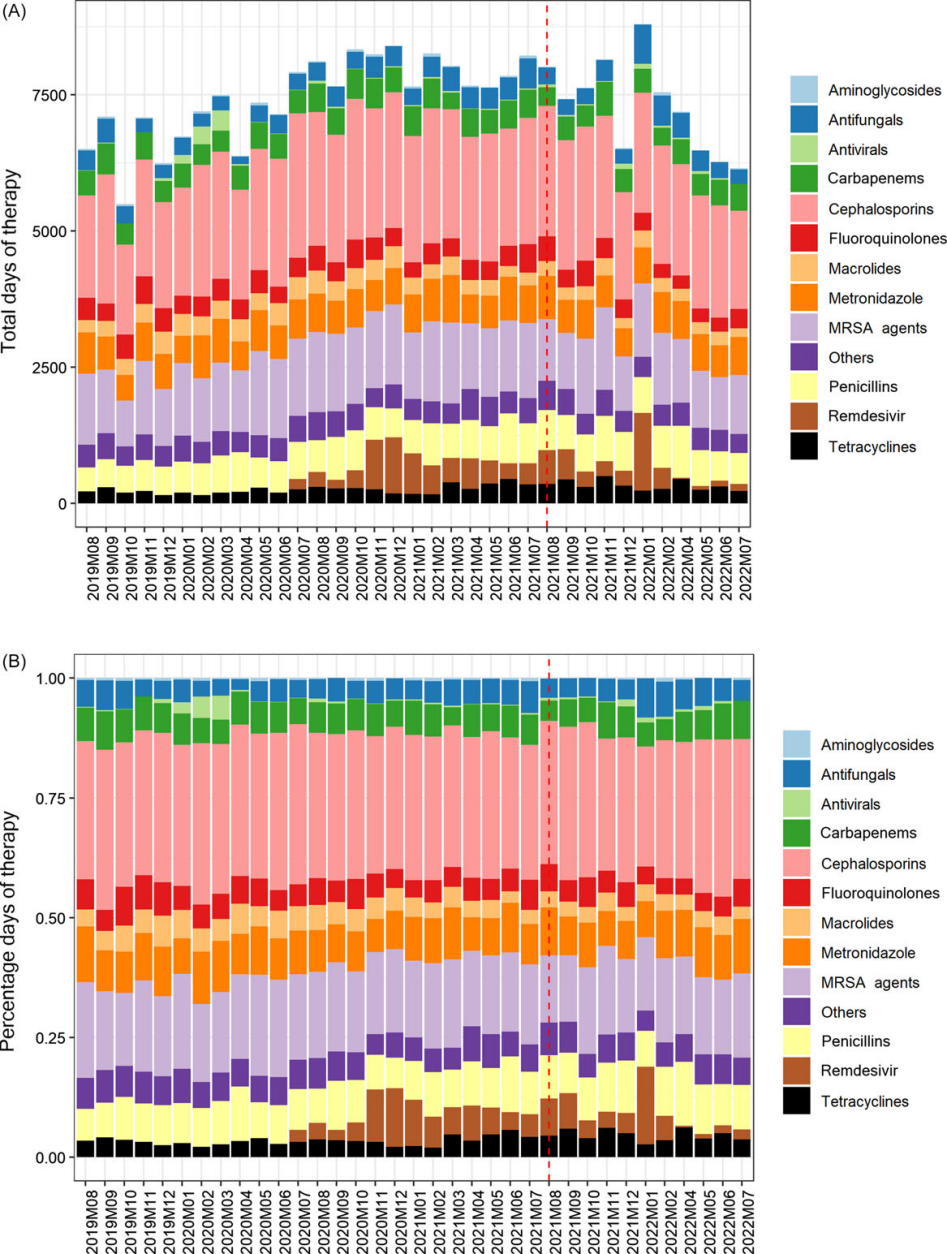
Total antibiotic usage by month and by antibiotic class, absolute (A) and percentage (B); intervention start shown as dashed red line.

Overall, hospital-wide antimicrobial use during the pre-implementation and post-implementation phases did not significantly differ, with the monthly median being 749 DOT/1,000 DP (IQR 744–762) before and 742 (IQR 725–763) after implementation (*p* = 0.7). Antimicrobial use outcomes specific to the thirteen medicine floors are displayed in Table 2. On the medicine floors, there was no significant difference in antimicrobial use pre- and post- implementation of handshake stewardship with 626 DOT/1,000 DP (IQR 523–712) vs 646 (IQR 530–719), *p* = 0.6. Figure 1 displays the interrupted time series for the thirteen medicine units. During the twenty-four months preimplementation, there was an increasing trend (*p* = 0.06), and post-implementation of handshake stewardship there was a decreasing trend in antimicrobial use (*p* = 0.3). There was no immediate change noticeable from the intervention (*p* = 0.4). However, in a post hoc supplementary analysis, there was a significant difference when the intervention effect was lagged by 3 months both in terms of the delayed “immediate” effect (*p* = 0.03) and for the post-implementation slope effect (*p* = 0.03). Trends in antimicrobial use per month for each of the 13 floors and groups of antimicrobials are displayed in Figures 2 and 3. Subgroup analysis excluding remdesivir showed the same trends (Supplementary Figure 1). Evaluating specific antimicrobial groups or agents, there were some significant differences in use before and after handshake stewardship was implemented, including a significant increase in tetracyclines (17 vs. 26, *p* < 0.001) and amoxicillin-clavulanate (11 vs. 15, *p* < 0.001). Use of macrolides (24 vs. 19, *p* = 0.001), meropenem (24 vs. 17, *p* = 0.007), and vancomycin (78 vs. 70, *p* = 0.002) were significantly decreased. Overall antimicrobial use was not significantly different pre- and post- intervention after applying an antimicrobial spectrum index to DOT/1,000 DP (3017 ASI/1,000 DP vs. 2,970 ASI/1,000 DP, *p* = 0.7). There was a marginal trend toward an increase in oral antimicrobials as measured by the dDOT/tDOT metric (0.32 vs. 0.34, *p* = 0.06).

**Table 2.** Antimicrobial days of therapy per 1000 days present

	Preimplementation	Post-Implementation	*p* value
	August 2019–July 2021	August 2021–July 2022	
Hospital wide, all units, all antimicrobials	749 [744,762]	742 [725,763]	0.7
Medicine units			
All antimicrobials	626 [523,712]	646 [530,719]	0.6
All antibacterials	567 [464,652)]	556 [448,659]	0.9
All antifungals	23 [12,38]	25 [14,40]	0.4
All antivirals	0 [0,3]	0 [0,4]	0.1
Medicine units, all antimicrobials			
Hospitalists-only floors	632 [581, 700]	583 [560, 661]	0.6
Mixed teaching and hospitalists floors			
Medicine/Cardiology floors	365 [292, 587]	362 [284, 423]	0.33
Medicine-only floors	658 [577, 739]	679 [613, 757]	0.1
Medicine units, antimicrobial groups			
Aminoglycosides	0 [0, 2]	0 [0,0]	<0.001
Carbapenems	35 [20, 53]	32 [18,50]	0.3
Cephalosporins	186 [153, 218]	180 [149, 213]	0.4
Fluoroquinolones	28 [16, 44]	25 [14, 39]	0.1
Macrolides	24 [14, 37]	19 [12, 26]	0.001
MRSA agents	105 [79, 129]	98 [71, 124]	0.06
Penicillin and derivatives	45 [31, 68]	56 [37, 76]	0.009
Tetracyclines	17 [9, 29]	26 [14, 42]	<0.001
Others	32 [22, 48]	33 [22, 42]	0.8
Medicine units, specific antimicrobials			
Amoxicillin-clavulanate	11 [6,17]	15 [7, 24]	<0.001
Cefepime	69 [52, 94]	67 [50, 88]	0.4
Ceftriaxone	78 [59, 99]	76 [60, 91]	0.2
Meropenem	24 [12, 37]	17 [10, 31]	0.007
Metronidazole	52 [31, 74]	53 [31, 76]	0.8
Nitrofurantoin	0 [0, 4]	3 [0, 7]	<0.001
Remdesivir	0 [0, 3]	0 [0, 12]	0.01
Vancomycin	78 [56, 101]	70 [47, 88]	0.002
Antibiotic spectrum index/1,000 d present	3017 [2439, 3474]	2970 [2357, 3528]	0.7
dDOT/tDOT	0.32 [0.28, 0.37]	0.34 [0.29, 0.39]	0.06

Note. dDOT, digestive days of therapy; tDOT, total days of therapy. Median (interquartile range). Penicillin and derivatives include aminopenicillins, oxacillin, amoxicillin-clavulanate, ampicillin-sulbactam, and piperacillin/tazobactam.

In-hospital mortality during the study period was unchanged pre- and post-intervention (0.02% (95% CI 0.02–0.03%) vs. 0.03% (95% CI 0.02–0.03%), *p* = 0.2) (see Table 3). The number of hospital onset *C. difficile* infection cases increased on the medicine floors in the post intervention group (monthly median 1.5 (IQR: 0.75–3) vs. 3.5 (IQR: 2.75–6.25), *p* < 0.001 (see Supplementary Figure 2). Median hospital length of stay increased post-intervention to 8.2 d (IQR: 2.7, 9.1) from 7.3 (IQR: 2.5, 8.1) d pre-intervention (*p* < 0.001) (see Supplementary Figure 3).

**Table 3.** Medicine units patient outcomes

	Preimplementation	Post-implementation	*P* value
	August 2019–July 2021	August 2021–July 2022	
Length of stay	7.3 [2.5, 8.1]	8.2 [2.7, 9.1]	<0.001
*C. difficile* infection, hospital-onset, monthly median	1.5 [0.75, 3]	3.5 [2.75, 6.25]	<0.001
Hospital mortality rate	0.02 [CI 0.02–0.03]	0.03 [CI 0.02–0.03]	0.2
Infectious diseases consults, monthly median	128 [117, 135]	128 [119, 137]	0.6

Note. Median (interquartile range); CI, confidence interval.

Response rates for the surveys sent for feedback about the handshake stewardship service were 28% (45/162) for medical residents and 43% (49/113) for hospitalists. Respondents indicated the frequency of contact with the handshake stewardship service was just right (88.5% and 88%) for medical residents and hospitalists, respectively. The preferred method of communication was in-person (66% for residents, 52% for hospitalists) followed by Epic® chat message (34% residents, 43% hospitalists). The full results of the surveys are available in the Supplementary Table 2. Residents and hospitalists responded that they felt the recommendations from handshake stewardship to be helpful always (54% and 48%), most of the time (40% and 38%), about half of the time (3% and 7%), or sometimes (3% and 7%), and no one answered the recommendations were never helpful. Free-text comments were overwhelmingly favorable.

## Discussion

After one year of performing handshake stewardship on the medicine floor teams at a large teaching hospital with an existing stewardship program, there have been some important shifts in patterns of overall antibiotic use. While there was not a significant decrease in overall antibiotic use immediately following the implementation of the intervention, there has been a reversal of a trend of overall increasing antibiotic use that was statistically significant when the intervention effect was lagged by 3 months. Additionally, there were significant decreases in the use of specific broad-spectrum agents such as vancomycin and meropenem. Another important shift in usage patterns observed was the trend toward an increasing preference for oral agents as opposed to intravenous agents with the dDOT/tDOT metric. This may suggest that there were shifts toward more appropriate antibiotic use.

Unlike prior descriptions of handshake stewardship services, we did not find a statistically significant change in overall antibiotic use, which could be attributed to several factors. First, the size of the hospital and number of providers impacted the reach of our service and could have diffused the effects of any changes in antibiotic use. A high intensity stewardship service implemented in five internal medicine units at a 400-bed community hospital in Toronto demonstrated a reduction in overall antibiotic use from 483 to 442 defined daily doses/1,000 patient days.^7^ Our study had 13 medicine units and >200 different providers some of whom were only on service a limited number of times over the year of the intervention thus impacting the reach of our intervention. Second, prior to this intervention, there was already an active antimicrobial stewardship program in place, and our starting antibiotic usage was slightly below the national average.^13^ Third, there is a significant proportion of antibiotic use driven by ID consult services, and handshake stewardship did not routinely review these antimicrobial regimens. The antibiotic use data was at the unit level; therefore, patients with ID consults were unable to be excluded from this outcome measurement. Fourth, the frequency of handshake stewardship rounds was only twice a week for medicine teaching services and once a week for hospitalist services. The Children’s Hospital of Colorado team, who pioneered handshake stewardship, started rounding three days per week but increased to five days per week after the first year and demonstrated a sustained decrease in overall antimicrobial use hospital wide after five years.^1^ The less frequent interaction in our study may have reduced the effect of the intervention. However, our acceptance rates compared favorably to prior studies describing this intervention. Seidelman et al. reported acceptance rates of 77.5% at one week,^6^ whereas Hurst et al. reported acceptance rates of 86% at the end of the workday.^14^

Safety measures in stewardship studies are crude, but we saw no change in in-hospital mortality in the pre- and post-intervention period. We did see an unexpected increase in both length-of-stay and *C. difficile* infection rates in the studied period. The former likely reflects the overall secular trend of increasing lengths of stay that has been observed nationally since the onset of the COVID-19 pandemic, while the latter likely reflects a significant change in the *C. difficile* testing protocol implemented in 2022 (see supplementary discussion).^15^

Stewardship interventions are difficult to generalize across different healthcare settings due to disparities in resources, tracking capabilities, baseline practice patterns, and regional microbiological profiles. While handshake stewardship is a resource-intensive intervention, the benefit of that upfront cost in effort is the inherent flexibility and adaptability of the intervention to specific contexts. Because of the personal and individual nature of the intervention, relationships between the stewardship team and multiple provider teams can be built and strengthened, which can then affect an overall shift in prescriber culture. These benefits are less tangible and difficult to track; however, the high intervention acceptance rate and enthusiastic provider survey feedback reflect these benefits. And indeed, following the initial enthusiastic response on the medicine floors, we expanded the intervention to the surgical floors and one of our ICUs. Based on the positive feedback from medical residents, we created a formal medical resident rotation, which has become popular. Handshake stewardship can also change with the institution—as patient populations, antibiograms, and institutional priorities shift, handshake stewardship teams can adapt in real time.

Limitations in this study stem in part from limitations in tracking capabilities. Only inpatient DOT were included in this analysis, and it is unknown whether the shorter durations recommended inpatient reliably translated to shorter outpatient durations. Second, our analysis was limited by waves of COVID surges that occurred through both the pre- and post-intervention periods, although we tried to account for this with some subgroup analysis excluding remdesivir. It has been shown that COVID surges are correlated with increased antibiotic use, which influenced our results.^16,17^ Third, we did not assess appropriateness or adherence with guidelines as part of this analysis.

In conclusion, adding a handshake stewardship team does endow additional benefits—both tangible, with shifts in antibiotic use patterns toward narrower and oral regimens, and less tangible benefits in changing overall hospital culture toward antibiotics by being a visible and accessible resource to help guide antibiotic use.

## Supporting information

Neuner et al. supplementary material Neuner et al. supplementary material

## Supplementary material

For supplementary material accompanying this paper visit http://doi.org/10.1017/ash.2023.465. click here to view supplementary material
