# Supplementary material for: Mixed methods evaluation of handshake antimicrobial stewardship on adult inpatient medicine floors
Source: Antimicrob Steward Healthc Epidemiol. 2023 Nov 16;3(1):e210. doi: 10.1017/ash.2023.465 (PMC10753471; doi:10.1017/ash.2023.465)
Supplement: Neuner et al. supplementary material [file S2732494X23004655sup001.pdf]

<u>Supplementary Appendix</u>

Supplementary Methods

- Interventions were input by the Handshake antimicrobial stewardship team after rounds using the institutional Epic Antimicrobial Stewardship Standard Operation Procedure. Definitions of Antimicrobial Stewardship Intervention types:
    - Allergy clarification: Use when documenting/updating reactions to antimicrobial allergies
    - Broaden coverage/address absent therapy: Use when empiric or pathogen-recommended antimicrobial therapy is recommended to be broadened due to insufficient coverage.
    - De-escalation: Use when antimicrobial therapy has been de-escalated either empirically or based on culture results.
    - Diagnostic-related: Use when recommending a diagnostic test (ex. MRSA nasal swab).
    - Discontinue: Use when an antimicrobial is discontinued and not replaced with another.
    - Dose optimization: Use when an antimicrobial dose and/or frequency is changed.
    - Duration of therapy: Use when a duration of therapy is extended.
    - Drug-interaction/Contraindicated: Use when a patient's antimicrobial is switched to an alternative safer regimen due to drug interaction or contraindication.

    - Monitoring-related: Use when recommending a non-therapeutic drug level lab (ex. Requesting additional susceptibilities from microbiology).
    - Route change intravenous to oral: Use when changing the same antibiotic from intravenous to oral or per tube.
- Interventions were reviewed for acceptance by the ID pharmacist the next business day and any recommendations that were implemented within 24 hours were counted as accepted.
- Partial acceptance was accounted for by splitting recommendations into sub-recommendations and marking each one as accepted or rejected (i.e. the recommendation was to switch from ceftriaxone to amoxicillin-clavulanate and shorten the duration by 3 days, but only the duration was shortened. This would be recorded as two separate recommendations with the former being rejected and the latter being accepted).
- Antimicrobial Spectrum Index – we calculated an antimicrobial spectrum index to describe potential shifts in broad to narrow spectrum agents. We utilized the methodology described by Dr. Gerber (reference 10) but modified and updated it to include newer antimicrobials as described in detail by Dr. Ilges (reference 11).
- dDOT/tDOT - as described in detail by Dr. Moehring, et al. (reference 12) we calculated digestive days of therapy (dDOT) over total days of therapy (tDOT) as a marker of intravenous to oral route conversions. dDOT as defined by NHSN was the number of days of therapy administered via an oral, tube, or per rectum route. tDOT was defined as the dDOT plus intravenous DOT.

Supplementary Tables

Table 1. Existing Antimicrobial Stewardship Program Details

ASP Strategy	Affected Antimicrobials
Prior Authorization	Amphotericin B, liposomal, Artesunate, Cabotegravir-Rilpivirine, Cefiderocol, Ceftazidime-avibactam, Ceftolozane-tazobactam, Chloramphenicol, Dalbavancin, Eravacycline, Imipenem-relebactam, Meropenem-vaborbactam, Tedizolid
72 hour Prospective audit and feedback	Ceftaroline, Ceftazidime, Colistin, Cidofovir, Daptomycin, Foscarnet, Maribavir, Quinupristin/dalfopristin, inhaled Ribavirin, Telavancin, Tigecycline
Restricted by Use Criteria	Aztreonam, Baloxavir, Letermovir, Remdesivir, SUBA-itraconazole
Guidelines and order sets	C. difficile Treatment Guidelines<br>COVID-19 Treatment Guidelines<br>Intra-abdominal Infection Guidelines<br>Pneumonia Treatment Guidelines<br>BioFire Pneumonia Panel Treatment Guidelines<br>Sexually Transmitted Infection Guidelines<br>Skin and Soft Tissue Infection Guidelines<br>Surgical Antimicrobial Prophylaxis Guidelines<br>Urinary Tract Infection for Hospitalized Patients Guidelines
Renal dosing policy	No automatic pharmacist renal dosing policy. However we utilize renal dose context on order entry to guide prescribers to the initial correct renal dose. In addition we have retrospective renal dosing clinical decision support rules that alert pharmacists 365 days/year when the dose ordered does not match the recommended dose.
Automatic IV to PO policy	There was a pharmacist Medication Therapy Management protocol for IV to PO antimicrobials in existence until December 2021 but given the low utilization of pharmacists using this automatic protocol (average 3 per month) compared to the number of IV to PO interventions being made and accepted outside of the confines of the protocol (average 55 per month) it was retired.

Table 2. Feedback Survey Results

Demographics		Medicine Residents N = 45	Hospitalists N = 49
Level of Training	PGY-1	16 (36%)	N/A
	PGY-2	12 (27%)	
	PGY-3	16 (36%)	
	Other	1 (1%)	
Clinical role	Hospitalists	N/A	41 (84%)
	Nurse Practitioner		8 (16%)
Years worked at BJH as Hospitalists		**N/A**	
< 1 year			6 (12%)
1-5 years			24 (49%)
5-10 years			8 (16%)
> 10 years			11 (23%)
Have you interacted with the Handshake ASP team in the past 5 months?		**Yes = 35 (78%)**	**Yes = 43 (88%)**

Survey Questions	Medicine Residents N = 35	Hospitalists N = 42*
How often do you contact the ASP team?	N/A	
Never		7 (17%)
< Once per month		5 (12%)
Once per month		12 (29%)
A few times per month		13 (31%)
Once per week		4 (10%)
Several times per week		1 (2%)
Is the frequency of contact adequate?		
Would prefer less frequently	1 (3%)	1 (2%)
Just right	31 (88.5%)	37 (88%)
Would prefer more frequently	3 (8.5%)	4 (10%)
How would you prefer to receive communications from the ASP team?		
In-person rounds	23 (66%)	22 (52%)
Epic chat message	12 (34%)	18 (43%)
Phone call	0	1 (2%)
Prefer zero communication	0	1 (2%)
How often do you find the recommendations from ASP to be helpful in patient care?		
Never	0	0
Sometimes	1 (3%)	3 (7%)

About half the time<br>Most of the time<br>Always	1 (3%)<br>14 (40%)<br>19 (54%)	3 (7%)<br>16 (38%)<br>20 (48%)
How often do you implement the ASP recommendations?<br>Never<br>Sometimes<br>About half the time<br>Most of the time<br>Always<br>No response	 0<br>1 (3%)<br>3 (9%)<br>19 (54%)<br>11 (30%)<br>1 (3%)	 0<br>3 (7%)<br>2 (5%)<br>21 (50%)<br>16 (28%)<br>0
How easy is it for you to get in touch with members of the ASP team?<br>Difficult<br>Neither easy nor difficult<br>Somewhat easy<br>Extremely easy<br>No response	 6 (17%)<br>10 (28.5%)<br>10 (28.5%)<br>7 (20%)<br>2 (6%)	 1 (2%)<br>8 (19%)<br>11 (26%)<br>22 (53%)<br>0
Would you be interested in rounding with the ASP team as an elective?<br>Definitely not<br>Probably not<br>Probably yes<br>Definitely yes<br>No response	1 (3%)<br>9 (25.5%)<br>13 (37%)<br>10 (28.5%)<br>2 (6%)	N/A
Would you be interested in any of the following activities or interventions?<br>Individual antibiotic use reports<br>Peer comparison reports for antibiotic use<br>Research collaboration with ASP<br>Hospitalists stewardship champion	N/A	 15 (30%)<br>22 (44%)<br>6 (12%)<br>7 (14%)

*While 43 survey participants responded they had interacted with the ASP team; only 42 participants completed the second part of the survey

Supplementary Figures

Figure 1. Total antibiotic usage by month and by antibiotic class excluding remdesivir, absolute (A) and percentage (B); intervention start shown as dashed red line

(A)

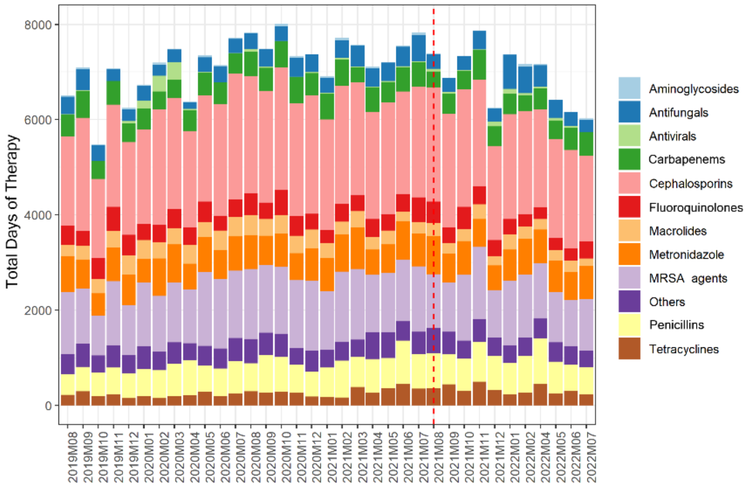

(B)

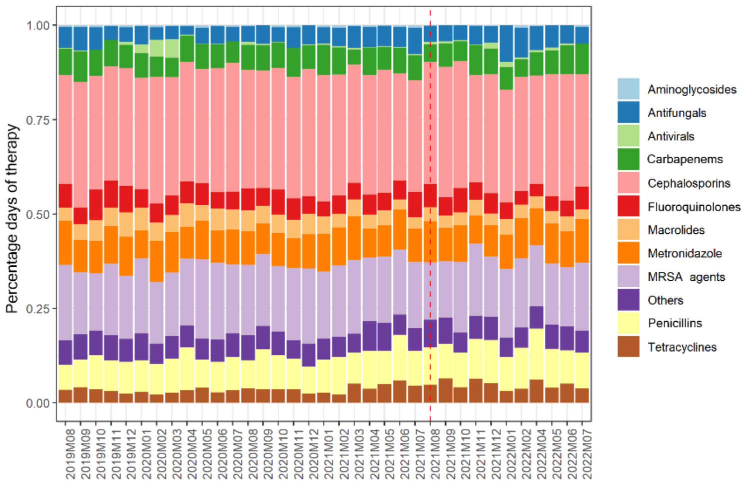

Figure 2. *C. dificile* infection rate by month; Intevention start red dashed line

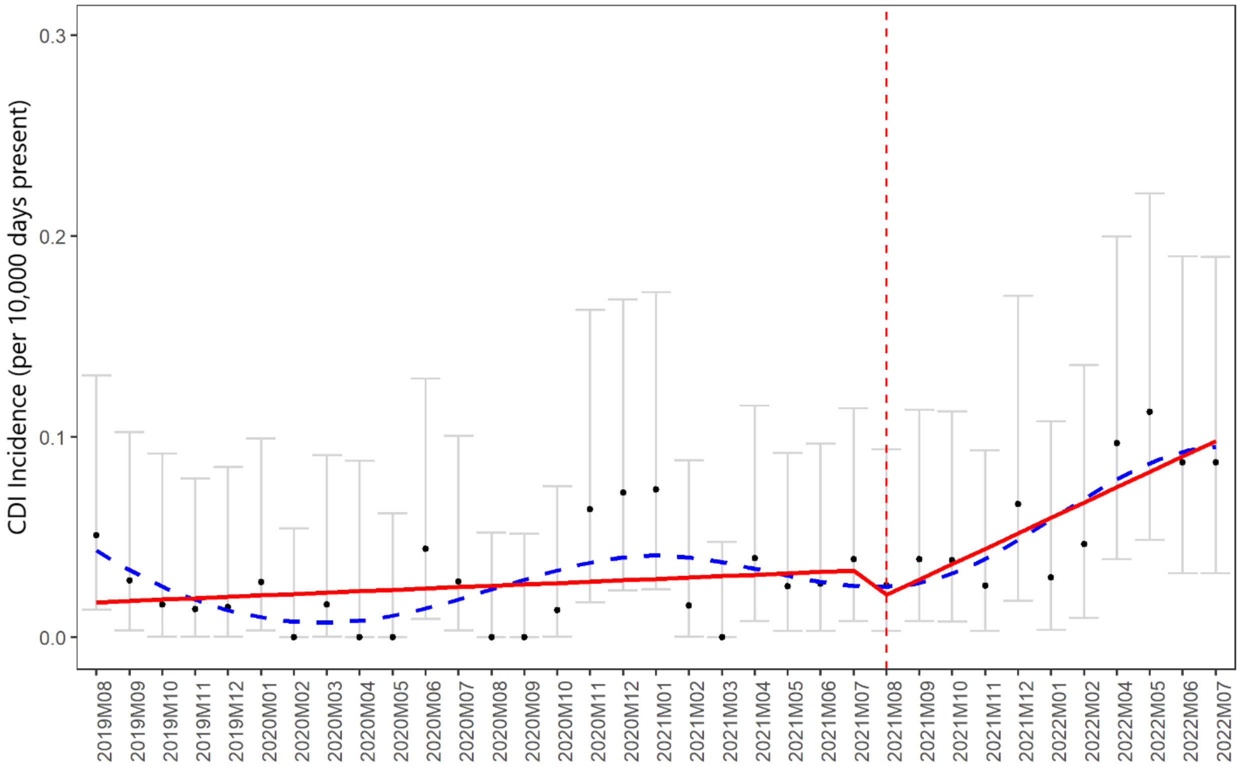

Figure 3. Length of stay by month; intervention start dashed red line; Omicron surge months labeled

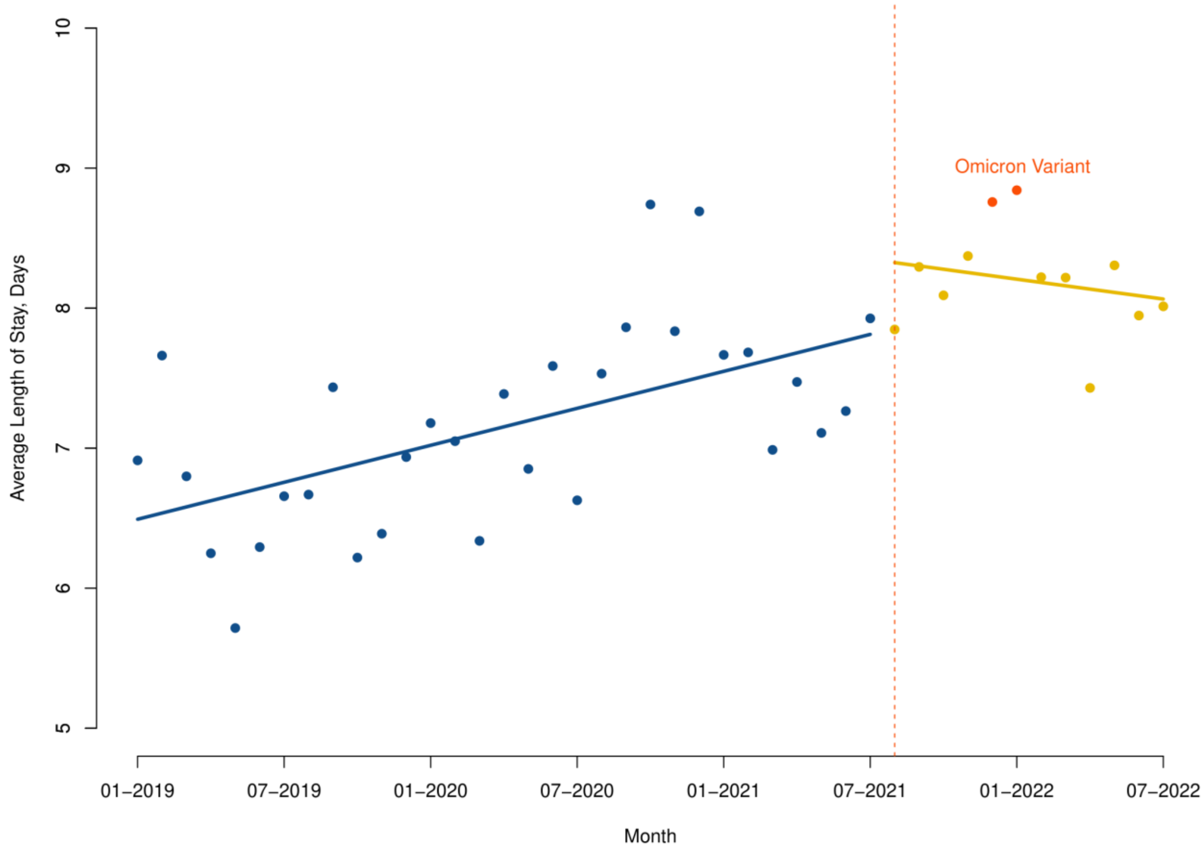

Supplementary Discussion

Interrupted time series analysis of our length of stay data reveals that the slope did decrease during the post-implementation period. There wasn't a significant immediate effect at intervention ($p = 0.162$) or with a 3 month lag ($p = 0.094$). Additionally, there is no statistically significant difference in slope ($p = 0.146$) immediately post-intervention. However, the difference in slope is statistically significant ($p = 0.039$) when lagged by 3 months – this may be supportive of the theory that the intervention may have had a dampening effect in the overall secular increase in length of stay that has been seen nationally since the beginning of the pandemic.

Our *C. difficile* infection rates increased and this was reflected in the ITS, but there were numerous confounders here including a fairly major change in our *C. difficile* testing protocol as discussed in the main text. Additionally, the trend in the slope was highly influenced by two above average post-intervention months - more data would be required for concrete conclusions to be drawn.
